# Coding SNPs analysis highlights genetic relationships and evolution pattern in eggplant complexes

**DOI:** 10.1371/journal.pone.0180774

**Published:** 2017-07-07

**Authors:** Alberto Acquadro, Lorenzo Barchi, Pietro Gramazio, Ezio Portis, Santiago Vilanova, Cinzia Comino, Mariola Plazas, Jaime Prohens, Sergio Lanteri

**Affiliations:** 1 University of Turin—DISAFA—Plant Genetics and Breeding, University of Turin, Largo Braccini 2, Grugliasco, Torino, Italy; 2 Instituto de Conservación y Mejora de la Agrodiversidad Valenciana, Universitat Politècnica de València, Camino de Vera 14, Valencia, Spain; 3 Instituto de Biología Molecular y Celular de Plantas, Consejo Superior de Investigaciones Científicas-Universitat Politècnica de València, Camino de Vera 14, Valencia, Spain; National Bureau of Plant Genetic Resources, Pusa India, INDIA

## Abstract

Brinjal (*Solanum melongena*), scarlet (*S*. *aethiopicum*) and gboma (*S*. *macrocarpon*) eggplants are three Old World domesticates. The genomic DNA of a collection of accessions belonging to the three cultivated species, along with a representation of various wild relatives, was characterized for the presence of single nucleotide polymorphisms (SNPs) using a genotype-by-sequencing approach. A total of 210 million useful reads were produced and were successfully aligned to the reference eggplant genome sequence. Out of the 75,399 polymorphic sites identified among the 76 entries in study, 12,859 were associated with coding sequence. A genetic relationships analysis, supported by the output of the *FastSTRUCTURE* software, identified four major sub-groups as present in the germplasm panel. The first of these clustered *S*. *aethiopicum* with its wild ancestor *S*. *anguivi*; the second, *S*. *melongena*, its wild progenitor *S*. *insanum*, and its relatives *S*. *incanum*, *S*. *lichtensteinii* and *S*. *linneanum*; the third, *S*. *macrocarpon* and its wild ancestor *S*. *dasyphyllum*; and the fourth, the New World species *S*. *sisymbriifolium*, *S*. *torvum* and *S*. *elaeagnifolium*. By applying a hierarchical *FastSTRUCTURE* analysis on partitioned data, it was also possible to resolve the ambiguous membership of the accessions of *S*. *campylacanthum*, *S*. *violaceum*, *S*. *lidii*, *S*. *vespertilio* and *S*. *tomentsum*, as well as to genetically differentiate the three species of New World Origin. A principal coordinates analysis performed both on the entire germplasm panel and also separately on the entries belonging to sub-groups revealed a clear separation among species, although not between each of the domesticates and their respective wild ancestors. There was no clear differentiation between either distinct cultivar groups or different geographical provenance. Adopting various approaches to analyze SNP variation provided support for interpretation of results. The genotyping-by-sequencing approach showed to be highly efficient for both quantifying genetic diversity and establishing genetic relationships among and within cultivated eggplants and their wild relatives. The relevance of these results to the evolution of eggplants, as well as to their genetic improvement, is discussed.

## Introduction

Eggplant, also known as brinjal eggplant or aubergine (*Solanum melongena* L., Solanaceae, 2n = 2x = 24), is cultivated worldwide and is one of the most important vegetable crops, being the second most important solanaceous crop grown for its fruit after tomato (*S*. *lycopersicum* L.) [1]. The bulk of eggplant production is concentrated in China, India, Iran, Egypt and Turkey, with Italy and Spain representing the most important European Union producers [1]. Because of its importance as a staple vegetable food in many countries from tropical and subtropical regions, eggplant is included with 34 other food crops in the Annex 1 of the International Treaty on Plant Genetic Resources for Food and Agriculture [2]. Eggplant berries are a source of dietary minerals as well as vitamins and other health-promoting metabolites such as anthocyanins and chlorogenic acid, with nutraceutical and anti-oxidant properties [3–6].

Brinjal eggplant selection and breeding over the years have been mainly focused on the improvement of fruit traits [7], such as size, weight, color, and shape [8,9], reduced prickliness, yield potential [10], and more recently organoleptic, nutritional and bioactive properties [11–14]. This has resulted in the development of a large number of eggplant varieties, whose fruit shape varies from flattened to elongated. However, like in many other domesticates, anthropogenic selection has resulted in a drastic reduction of the genetic variation across eggplant genome, due to both the genetic bottleneck resulting from the sampling process of a limited number of wild plants chosen for domestication [15] and the migration of a limited number of genotypes from the primary to the secondary centers of domestication [16]. The eggplant inter-fertile cultivated species as well as crop wild relatives (CWRs) are a source of variation for many traits of interest and represent an obvious target to aid eggplant improvement; however, to date, their potential use has largely remained unexploited [17].

Unlike tomato (*S*. *lycopersicum*), potato (*S*. *tuberosum* L.) and pepper (*Capsicum* spp.), eggplant is native to the Old World and was independently domesticated from *S*. *insanum* L. in the Indian subcontinent and in China [16,18], with a possible additional and independent center of domestication in the Philippines [19]. Besides *S*. *melongena*, two other eggplant species are commonly grown in sub-Saharan Africa [20], the scarlet eggplant (*S*. *aethiopicum* L.) and the gboma eggplant (*S*. *macrocarpon* L). Both species can be inter-crossed with brinjal eggplant producing hybrids with intermediate fertility [21]. The three cultivated eggplants belong to the *Leptostemonum* clade and to a species-rich subclade composed exclusively of Old World taxa (the Old World clade sensu [22,23]) from Africa, Australia, and Asia (including Eurasia and the Middle East).

Scarlet eggplant (*S*. *aethiopicum* L.) is an important vegetable in Central and West Africa, but it is also cultivated in the Caribbean and Brazil as well as in some areas of South Italy [24]. It is a hypervariable species and includes hundreds of local varieties [25] clustered in four main cultivar groups: Aculeatum, Gilo, Kumba and Shum, which are completely inter-fertile [21]. The four cultivar groups are differently exploited, since Aculeatum is used as ornamental, Gilo for its fruits, Kumba for both fruits and leaves, while Shum for its leaves [25,26]. Gboma eggplant (*S*. *macrocarpon* L.) is less widespread in cultivation, although the species represents a major vegetable in some countries like Benin and in the rain forest regions of Coastal Africa and Congo River [27]. It is also a morphologically variable species and it is grown for its fruits, leaves or both [25,26]. The high variability within both scarlet and gboma eggplants has been recently confirmed by Plazas et al. [21], whom by applying conventional descriptors as well as the high-throughput Tomato Analyzer phenomics tool characterized a wide set of accessions of both cultivated species as well as from the scarlet eggplant wild ancestor *S*. *anguivi* Lam., *S*. *aethiopicum-S*.*anguivi* intermediate forms, and the gboma eggplant wild ancestor *S*. *dasyphyllum* Schumach. & Thonn. Each of the three cultivated eggplants together with their wild ancestors and the closest wild relatives are commonly referred to as the brinjal, scarlet and gboma eggplant complexes [10,18,21,26].

Wild relatives of cultivated eggplants, which are well adapted to grow in a wide range of conditions, from desert to swampy areas and environments with wide ranges of temperatures, are a source of useful traits for eggplants breeding. Unfortunately the latter remain largely unexploited and a limited number of reports on the use of the variation available in the wild species has been reported [17,20,28] while, to our knowledge, no modern commercial varieties of eggplants carry introgression from wild species.

In brinjal eggplant and related species the delimitation of biologically meaningful genepools is challenging due to limited crossability data reported in literature [29], as well as to the extremely large number of potential genepool members. By taking into account both relatedness, as measured by phylogenetic analyses and available data on crossability, recently Syfert et al. [30] suggested the inclusion of one species (*S*. *insanum*) in the primary genepool (GP1), forty-eight species with which eggplant can be inter-crossed with varying degrees of difficulty in GP2, and three wild and weedy species native to the New World in GP3, i.e. *S*. *sisymbriifolium* Lam., *S*. *torvum* Sw. and *S*. *viarum* Dunal, with which only highly sterile hybrids can be obtained through embryo rescue or are not obtainable.

The great advances in next generation sequencing (NGS) technologies, with rapid increases in data volumes and quality combined with reducing costs, have provided breeders with a wide array of genomic tools which facilitate the characterization of germplasm collections and allow to gain a better understanding of how the genome contributes to the diversity detected at phenotypic level [31]. Single nucleotide polymorphisms (SNPs) represent the most frequent type of genetic polymorphism and have become the marker of choice for many applications in plant biology, conservation and breeding [32].

Here we report a genotype by sequencing (GBS) approach based on reducing genome complexity to detect SNPs polymorphisms in a set of seventy-six accessions of species belonging to the brinjal, gboma and scarlet eggplant complexes, which include taxa included in the *S*. *melongena* primary, secondary and tertiary genepools. Our main goal was to assess, using a high-throughput genotyping technique, the genetic relationships within and between the genepools of the brinjal eggplant (*S*. *melongena*) and the two other cultivated eggplants, namely the scarlet (*S*. *aethiopicum*) and gboma (*S*. *macrocarpon*) eggplants. Apart from cultivated accessions, we also included in the study accessions of close wild relatives of the three crops, as well more distant species from the tertiary genepool species. The information obtained will be of great relevance for clarifying the relationships among cultivated and wild eggplants and will be useful to breeders using wild species for eggplant breeding

## Material and methods

### Plant materials

A total of 76 accessions, including 16 entries of *S*. *melongena* from Asian and European origin, 30 of *S*. *aethiopicum* belonging to the four varietal groups (Aculeatum, Gilo, Kumba and Shum) plus intermediate forms between *S*. *aethiopicum* and *S*. *anguivi*, five of *S*. *macrocarpon*, and 25 accessions of 14 wild species were used for the present study (Table 1). Among the 16 entries of brinjal eggplant, two of them are doubled haploids (S. melongena_10 and S_melongena_12) obtained by anther culture [33]. Also, four brinjal eggplant entries come from two original sources (entries S. melongena_1 and S. melongena_2 from the original source MEL1; and accessions S. melongena_6 and S. melongena_7 from the original source MEL5) (Table 1). Among the wild relatives are included the putative ancestors of brinjal eggplant (*S*. *insanum*), scarlet eggplant (*S*. *anguivi*), and gboma eggplant (*S*. *dasyphyllum*) [34–36], as well as eight other wild species from Old World origin (*S*. *campylacanthum* Hochst. ex A. Rich, *S*. *incanum* L., *S*. *lichtensteinii* Willd., *S*. *lidii* Sunding, *S*. *linnaeanum* Hepper & P.-M.L. Jaeger, *S*. *tomentosum* L., *S*. *vespertilio* Aiton, and *S*. *violaceum* Ortega), and three native to the New World (*S*. *elaeagnifolium* Cav., *S*. *sisymbriifolium*, and *S*. *torvum*) [30,37]. All these materials are conserved in the germplasm collection maintained at Universitat Politècnica de València (Valencia, Spain).

**Table 1:** Plant materials used including taxon, accession name, accession code used in the present work, country of origin and fruit shape and predominant colour.

Taxon and accession	Code	Country of origin	Fruit shape^a^	Predominant fruit colour^b^
*S*. *aethiopicum* L. gr. Aculeatum				
MM457	S. aethiopicum aculeatum_1	Japan	1	1.3
UPV29803	S. aethiopicum aculeatum_2	China	1	1.2
RNL0187	S. aethiopicum aculeatum_3	Burkina Faso	1	1.2
MM1483	S. aethiopicum aculeatum_4	Ghana	1	1.3
*S*. *aethiopicum* L. gr. Gilo				
BBS151A	S. aethiopicum gilo_1	Ivory Coast	7	1.1
IVIA026	S. aethiopicum gilo_2	Unknown	7	1.2
RARE_PLANTS_GILO	S. aethiopicum gilo_3	Unknown	3	1.3
RNL0252	S. aethiopicum gilo_4	Ghana	3	1.2
UPV29014	S. aethiopicum gilo_5	Unknown	5	1.2
RNL0395	S. aethiopicum gilo_6	Liberia	3	1.1
RNL0288	S. aethiopicum gilo_7	Ghana	5	2
BBS181A	S. aethiopicum gilo_8	Ivory Coast	1	1.3
BBS147G	S. aethiopicum gilo_9	Ivory Coast	1	1.3
BBS140B	S. aethiopicum gilo_10	Ivory Coast	3	1.2
BBS159B	S. aethiopicum gilo_11	Ivory Coast	5	1.1
BBS142A	S. aethiopicum gilo_12	Ivory Coast	5	1.2
AN05	S. aethiopicum gilo_13	Angola	3	1.1
*S*. *aethiopicum* L. gr. Kumba				
INRA_4	S. aethiopicum kumba_1	Senegal	1	1.1
MM1207	S. aethiopicum kumba_2	Mali	1	1.1
BBS111	S. aethiopicum kumba_3	Ivory Coast	1	2
BBS110	S. aethiopicum kumba_4	Ivory Coast	1	1.1
*S*. *aethiopicum* L. gr. Shum				
RNL0022	S. aethiopicum shum_1	Benin	3	1.3
RNL_0340	S. aethiopicum shum_2	Zimbabwe	1	1.2
*S*. *aethiopicum* L.*-S*. *anguivi* Lam. intermediate				
BBS116	S. aethiopicum-anguivi_1	Ivory Coast	3	1.3
BBS192E	S. aethiopicum-anguivi_2	Ivory Coast	5	1.2
BBS148D	S. aethiopicum-anguivi_3	Ivory Coast	3	1.1
BBS131C	S. aethiopicum-anguivi_4	Ivory Coast	3	1.1
BBS184	S. aethiopicum-anguivi_5	Ivory Coast	3	1.1
BBS180A	S. aethiopicum-anguivi_6	Ivory Coast	5	1.1
BBS114	S. aethiopicum-anguivi_7	Ivory Coast	5	1.2
*S*. *anguivi* Lam.				
ANG1	S. anguivi_1	Ivory Coast	3	1.1
ANG2	S. anguivi_2	Ivory Coast	3	1.3
*S*. *campylacanthum* Hochst. ex A. Rich				
CAM5	S. campylacanthum_1	Tanzania	3	1.2
CAM6	S. campylacanthum_2	Kenya	3	1.2
CAM8	S. campylacanthum_3	Tanzania	3	1.2
*S*. *dasyphyllum* Schumach. & Thonn.				
DAS1	S. dasyphyllum_1	Uganda	1	1.2
*S*. *elaeagnifolium* Cav.				
ELE1	S. elaeagnifolium_1	Senegal	3	1.2
ELE2	S. elaeagnifolium_2	Greece	3	1.2
*S*. *incanum* L.				
MM577	S. incanum_1	Israel	5	1.2
*S*. *insanum* L.				
INS1	S. insanum_1	Sri Lanka	5	1.2
INS2	S. insanum_2	Sri Lanka	3	1.2
INS3	S. insanum_3	Japan	3	1.2
*S*. *lichtensteinii* Willd.				
LIC1	S. lichtensteinii_1	South Africa	3	1.3
LIC2	S. lichtensteinii_2	Iran	3	1.1
*S*. *lidii* Sunding				
LID1	S. lidii_1	Spain	3	1.3
LID2	S. lidii_2	Spain	3	1.3
*S*. *linnaeanum* Hepper & P.-M.L. Jaeger				
LIN1	S. linnaeanum_1	Spain	3	1.3
LIN3	S. linnaeanum_2	Tunisia	3	1.3
*S*. *macrocarpon* L.				
MM1558	S. macrocarpon_1	Malaysia	1	2
BBS168	S. macrocarpon_2	Ivory Coast	1	2
BBS117	S. macrocarpon_3	Ivory Coast	1	1.3
BBS171B	S. macrocarpon_4	Ivory Coast	1	2
BBS178	S. macrocarpon_5	Ivory Coast	5	1.2
*S*. *melongena* L.				
MEL1_2	S. melongena_1	Ivory Coast	5	2
MEL1_3	S. melongena_2	Ivory Coast	5	2
MEL2	S. melongena_3	Ivory Coast	5	7
MEL3	S. melongena_4	Ivory Coast	7	1.2
MEL4	S. melongena_5	Sri Lanka	3	7
MEL5_2	S. melongena_6	Sri Lanka	7	7
MEL5_5	S. melongena_7	Sri Lanka	7	7
MEL6	S. melongena_8	Sri Lanka	7	7
AN-S-26	S. melongena_9	Spain	5	7
DH_AN-S-26	S. melongena_10	Spain	5	7
MM1597	S. melongena_11	India	9	1.2
DH_ECAVI	S. melongena_12	Breeding line	7	8
H15	S. melongena_13	Spain	5	7
A0413	S. melongena_14	Unknown	1	2
ASI-S-1	S. melongena_15	China	1	8
IVIA371	S. melongena_16	Spain	5	7
*S*. *sisymbriifolium* Lam.				
SIS1	S. sisymbriifolium_1	Unknown	3	1.2
SIS2	S. sisymbriifolium_2	Unknown	5	1.3
*S*. *tomentosum* L.				
TOM1	S. tomentosum_1	South Africa	3	1.3
*S*. *torvum* Sw.				
TOR2	S. torvum_1	Sri Lanka	3	1.2
TOR3	S. torvum_2	Unknown	3	1.3
*S*. *vespertilio* Aiton				
VES2	S. vespertilio_1	Spain	3	1.3
*S*. *violaceum* Ortega				
VIO1	S. violaceum_1	Sri Lanka	3	1.2

^a^Fruit shape according to the following scale: 1 = broader than long; 3 = as long as broad; 5 = slightly longer than broad; 7 = twice as long as broad; 8 = three times as long as broad; 9 = several times as long as broad.

^b^Fruit predominant colour when the fruit is physiologically immature according to the following categories, in which the green colour (1) has been subdivided into three subcategories: 1.1 = clear green; 1.2 = intermediate green; 1.3 = dark green; 2 = milk white; 3 = deep yellow; 4 = fire red; 5 = scarlet red; 6 = lilac grey; 7 = purple; 8 = purple black; 9 = black.

### Library construction and sequencing

DNA was extracted following a modified CTAB method [38] as indicated elsewhere [39]. Library construction (11/2015) was performed as proposed in Peterson et al. [40] and modified as in Acquadro et al. [41], by using a HindIII-MseI enzyme combination and adding a final biotin/streptavidin-coated beads based purification step. Quality, quantity and reproducibility of libraries were assesed on a Bioanalyzer instrument (DNA High Sensitivity chip) as well as qPCR. On the basis of the quantitation, DNA libraries were pooled and sequenced on Illumina HiSeq 2500 platform (Illumina Inc., San Diego, CA, USA), following the manufacturer protocol using 100SE chemistry.

### Sequence analysis

Raw reads were analyzed with Scythe (https://github.com/vsbuffalo/scythe) for filtering out contaminant substrings and Sickle (https://github.com/najoshi/sickle), which allows to remove reads with poor quality ends (Q<30). Illumina reads were de-multiplexed on the basis of the Illumina TruSeq index. Alignment to the reference eggplant genome [42,43] was carried out using BWA aligner [44] (i.e., mem command) with default parameters and avoiding multiple-mapping reads. SNP mining was conducted by adopting a Samtools-based pipeline [45]. Homozygous/heterozygous SNP/Indel calls were considered only with phred-scaled genotype likelihood equal zero. A catalog of candidate high quality SNPs was produced. Relationships among the genotypes were computed using: i) whole genome, and ii) coding (within exons) SNP/indel datasets. The proportion of heterozygous SNPs for each genotype was estimated by the ratio of total number of heterozygous SNPs and all the detected SNPs (excluding missing SNPs) as well as the ratio of the number of heterozygous SNPs in coding regions and all the detected SNPs in coding regions.

### Genetic relationships analysis and population structure

SNP data were coded according to the number of occurring polymorphisms, being assigned a 0 if they showed the homozygous reference type, a 1 if the variant occurred in one chromosome and a 2 if the variant was present in both chromosomes. Genetic similarities between pairs of entries were quantified by the Dice similarity index [46] as 2*m*^*+*^/(2*m*^*+*^ + *m*^*-*^), were *m*^*+*^ is the number of matches (1–1 and 2–2) and *m*^*-*^ is the number of mismatches (0–1, 0–2 and 1–2). Genetic relationships were described by using both the unweighted pair-group arithmetic mean (UPGMA) method with 1,000 bootstraps, and principal coordinate analysis (PCoA) by means of Past 3.14 software [47]. A co-phenetic matrix was also produced using the hierarchical cluster system, by means of the COPH (cophenetic values) routine, and correlated with the original distance matrix, in order to test for associations between clusters and the similarity matrix.

*FastSTRUCTURE* [48] was used to estimate the number of sub-populations in the panel, applying the admixture model for the ancestry of individuals and correlated allele frequencies. A hierarchical *FastSTRUCTURE* analysis [49] was also applied on accessions which clustered in sub-group 1 and subgroup 4 following UPGMA analysis as well as on the set of all the remaining. The program was run with default setting using simple prior to obtain a reasonable range of values for the number of populations (K), *FastSTRUCTURE* was executed for multiple values of K (K = 1–9). The script chooseK.py [48] was then used to infer the most likely number of populations.

## Results and discussion

### Sequencing and SNPs identification

A total of 225 million single reads were produced. About 94% of raw reads contained the expected restriction site overhang, along with discriminating inline barcodes. The average number of successfully de-multiplexed reads per sample was 2.7 M, with a standard deviation of 1.5 M (S1 Fig). Sequences were trimmed and quality cleaned to 210 million of useful reads (6.2% discarded). The latter were then aligned to the recently produced reference eggplant genome [42,43] and close to 100% of reads were successfully mapped to single regions (no multiple mapping was permitted). Mapped sequences showed an extensive coverage alongside the 12 chromosomes (data not shown).

In all, 75,399 polymorphic sites were identified among the 76 lines in study. Overall, all the *S*. *melongena* accessions, together with the three *S*. *insanum* accessions, showed a reduced level of polymorphism (on average 2.47 and 4.75% respectively) when aligned to the reference genome. On the other hand the frequency of polymorphic SNPs ranged from 10.62 to 24.32% in the other entries (S1 Table).

**Fig 1:**
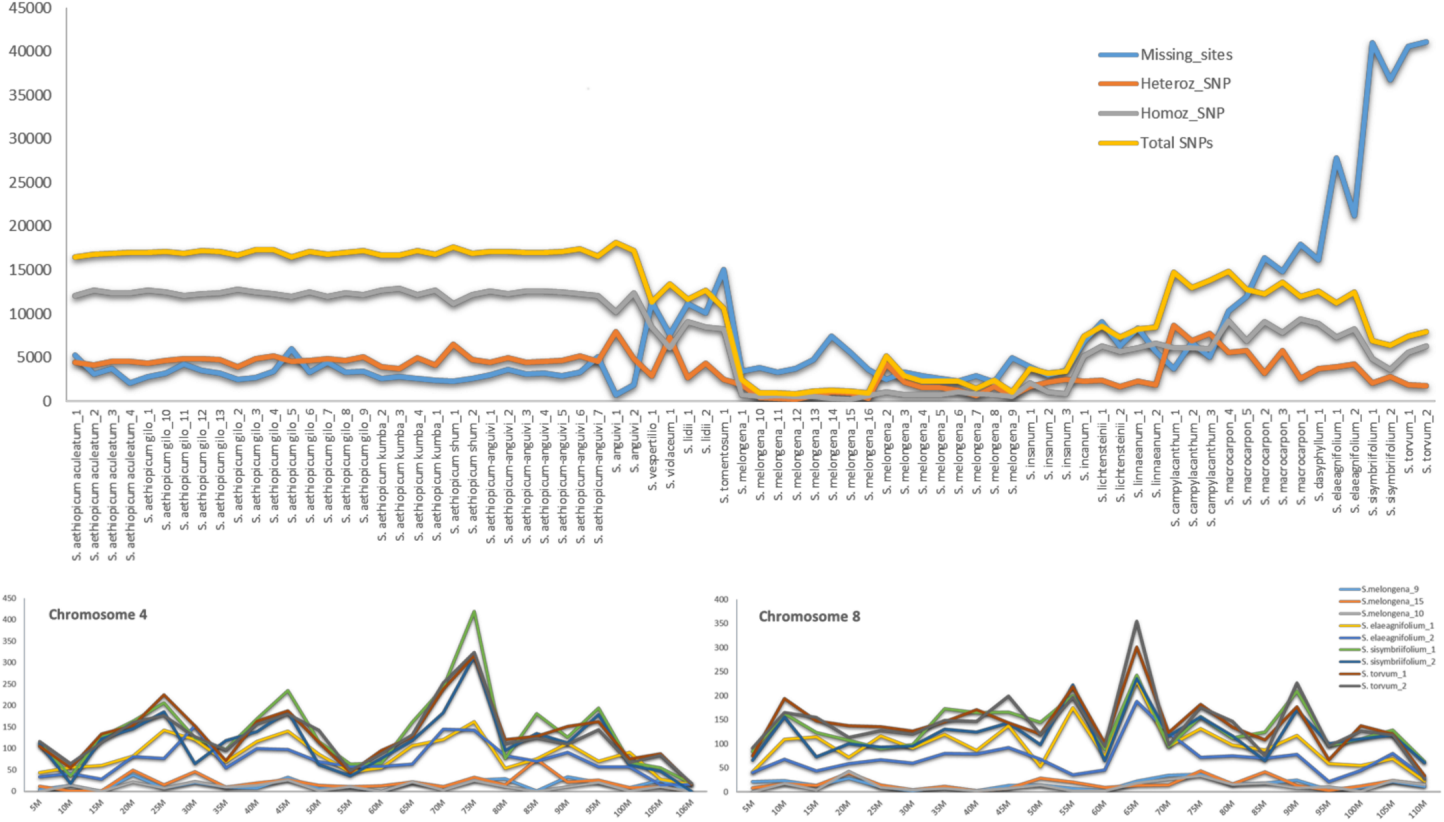
**SNP numbers and distribution:** A) Plot of the number of SNPs (total, homozygous and heterozygous) and missing sites observed within the collection of 76 *Solanum* accessions; B) The distribution of missing sites along two eggplant chromosomes (i.e.: chr4 and chr8). The trend lines track missing sites in three *Solanum melongena* and six accessions of American origin (*S*. *elaeagnifolium*, *S*. *sisymbriifolium and S*. *torvum)*.

*Solanum melongena* is a largely autogamous species [20], thus its low level of heterozygosity (on average 1.66%) is coherent with the expectation that germplasm accessions and non-hybrid varieties should be highly homozygous (Fig 1, S1 Table). Interestingly, the two *S*. *melongena* varieties (S. melongena_10, S. melongena_12, Fig 1, S1 Table), which are the result of diploidization of haploid plants obtained through anther culture, displayed some heterozygosity (<0.5%). This might be due to somaclonal variation, which is manifested as cytological abnormalities, sequence change, and gene activation and silencing which occur through the ‘in vitro’ culture process and that provides evidence that DNA modifications occur more frequently in ‘in vitro’ cultivated than in seed-grown plants [50]. However, it might be also a consequence of SNPs mapping on paralog genes since, similarly to tomato, potato and pepper, also eggplant the genome carries signs of the “T” triplication occurred during Solanaceae evolution [42], or being the results of some mapping artifacts. This would suggest that the heterozygosity detected in the rest of *S*. *melongena* accessions would be overestimated by almost one third.

The two other cultivated eggplant species (i.e., *S*. *aethiopicum* and *S*. *macrocarpon*) showed, on average, higher heterozygosity than *S*. *melongena*, ranging from 4.52 to 9.53% (Fig 1, S1 Table). This might be a consequence of their higher allogamy and the more limited breeding efforts for stabilizing phenotypic and yield-related traits. Low heterozygosity was also observed in the wild *S*. *insanum*, *S*. *lichtensteinii* and *S*. *linnaeanum* (< 3.5%), while higher values, over 10%, were observed in the wild species *S*. *campylacanthum*, *S*. *anguivi* and *S*. *violaceum*.

Some missing data were observed in *S*. *melongena* and *S*. *aethiopicum* (ranging from 2.71% to 9.87%), some accessions showed a medium-high level of missing data (e.g., *S*. *macrocarpon* 19% on average), while others showed a surprisingly high number of missing data (up to 54.5%, 54.43 and 36.88, in *Solanum torvum*, *S*. *sisymbriifolium* and *S*. *elaeagnifolium*, respectively). This might be explained by the fact that these latter species are native to the New World [30,37] and in consequence have a more distant common ancestor, and greater evolutionary divergence. Missing data were distributed on the different eggplant chromosomes; however, by adopting a five million bases sliding window analysis, some hot spot regions were highlighted (Fig 1). The filtering of the whole SNP dataset for the sites present in CDS regions granted 12,859 SNPs. The latter were used for all the subsequent analyses, since the relative number of missing data was lower in the coding dataset (3% on average) than in the whole dataset (10% on average, Fig 1, S1 Table). As an example the percentage of missing data of South American accessions (*S*. *elaeagnifolium*, *S*. *sisymbriifolium* and *S*. *torvum–*S1 Table) was lowered from about 46.1% to 15.1%, thereby increasing the resolution power of our analyses.

### Genetic relationships analysis and population structure

**Fig 2:**
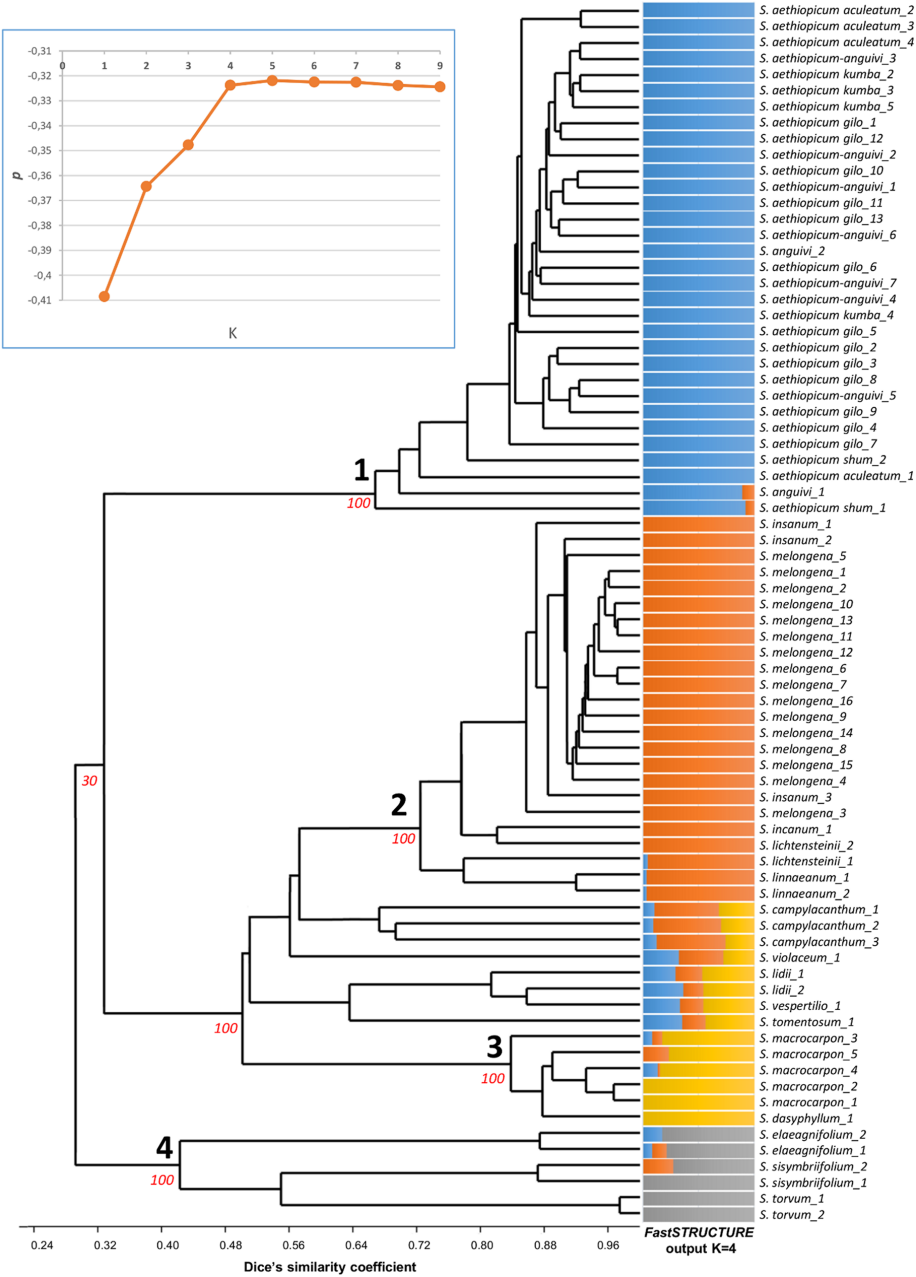
The genetic architecture of the full germplasm panel: Consensus UPGMA dendrogram and *FastSTRUCTURE* output at K = 4. Bootstrap values (%) for the main nodes are reported in red. Each entry is represented by a vertical line representing sub-group 1 (blue) sub-group 2 (orange), sub-group 3 (yellow) and sub-group 4 (grey). The box reports the Probabilities (p) plots derived from the *FastSTRUCTURE* analysis of genotypic data with K values from 1 to 9.

The UPGMA-based dendrogram and the output of *FastSTRUCTURE* [48] analysis (Fig 2) show the genetic relationships between the 76 accessions. Both, as well as the K analysis (Fig 2 box), suggest a population structure comprising four sub-groups. Each entry was fingerprinted and the co-phenetic correlation coefficient (r-value) between the Dice data matrix and the co-phenetic matrix was 0.978, highlighting a very good fit between the dendrogram clusters and the similarity matrices from which they were derived, indicating that the UPGMA method is suitable for the interpretation of our data. The fact that the sister entries S. melongena_1 and S. melongena_2, which are derivatives from the original source MEL1 cluster together in the dendrogram, and the same occurs for accessions S. melongena_6 and S. melongena_7, which derive from MEL5 provide a confirmation that the analysis is congruent.

According to the level of membership provided by *FastSTRUCTURE* [48], sub-group 1 (blue) includes all the accessions of scarlet eggplant (*S*. *aethiopicum*) and *S*. *anguivi*, which on the basis of previous studies has been reported to be its wild ancestor [30,34,51]. Sub-group 2 (orange) includes members of the brinjal eggplant complex [52,53], among which the most genetically related accessions of *S*. *melongena* and its wild progenitor *S*. *insanum*, the accession of *S*. *incanum*, and the two of both *S*. *lichtensteinii* and *S*. *linneanum*. Sub-group 3 (yellow) includes the five accessions of gboma eggplant (*S*. *macrocarpon*) and the one *S*. *dasyphyllum*, which is its wild progenitor [30,35]. Sub-group 4 (grey) includes the accessions of the New World species, which form part of the tertiary genepool of brinjal eggplant [30]. Finally, the remaining accessions of *S*. *campylacanthum*, *S*. *violaceum*, *S*. *lidii*, *S*. *vespertilio* and *S*. *tomentosum* had ambiguous membership and were thus classified as admixed, as their level of membership to a single group was lower than 70% (Fig 2). With the goal to provide insight into the complex relationships of the germplasm used, and to detect additional sub-population structure, a hierarchical *FastSTRUCTURE* analysis was applied by running *STRUCTURE* on partioned data, i.e. on accessions which clustered in sub-group 1 and sub-group 4 following UPGMA analysis, as well as on the remaining materials (S2 Fig). The hierarchical *FastSTRUCTURE* analysis for the scarlet eggplant complex revealed that the optimal number of populations was obtained at K = 2, and that the accessions of *S*. *aethiopicum* and *S*. *anguivi*, included in the UPGMA subgroup 1, share a common genepool. For the brinjal eggplant and gboma eggplant complexes group, the hierarchical *FastSTRUCTURE* analysis suggests that four populations are present. In this set of accessions K = 2 separates the brinjal eggplant *S*. *melongena* and its close relatives *S*. *insanum*, *S*. *incanum*, *S*. *lichtensteinii* and *S*. *linnaeanum* [18] from the gboma eggplant *S*. *macrocarpon* and its wild ancestor *S*. *dasyphyllum* [35] together with the Canary Islands endemisms *S*. *lidii* and *S*. *vespertilio* and the related South African *S*. *tomentosum* [23,54,55], while *S*. *campylacanthum* and *S*. *violaceum* appear as an admixture (S2 Fig). At K = 3 the *S*. *lidii*, *S*. *vespertilio*, *S*. *tomentosum* and *S*. *violaceum* are separated from the gboma eggplant. Finally, at the optimal K = 4, *S*. *campylacanthum* accessions group separately, while *S*. *incanum*, *S*. *lichtensteinii* and *S*. *linnaeanum* appear as an admixture of *S*. *melongena*/*S*. *insanum* and *S*. *campylacanthum* (S2 Fig). This might be a consequence of gene flow among them or the result of the recent speciation from a common ancestor or both. These species are phylogenetically closely related but at present are distributed in different geographical areas [18]; this suggests that presumably they evolved from a common ancestor for adaptation to different niches, which might difficult gene flow. The hierarchical *FastSTRUCTURE* analysis of the New World species recognized at K = 2 two populations, one of which included *S*. *elaeagnifolium* while the other both *S*. *sysimbriifolium* and *S*. *torvum*. However, at the optimal K = 3, the latter was further splitted in two genetically differentiated genepools, each including one of the two species.

### PCoA analyses

**Fig 3:**
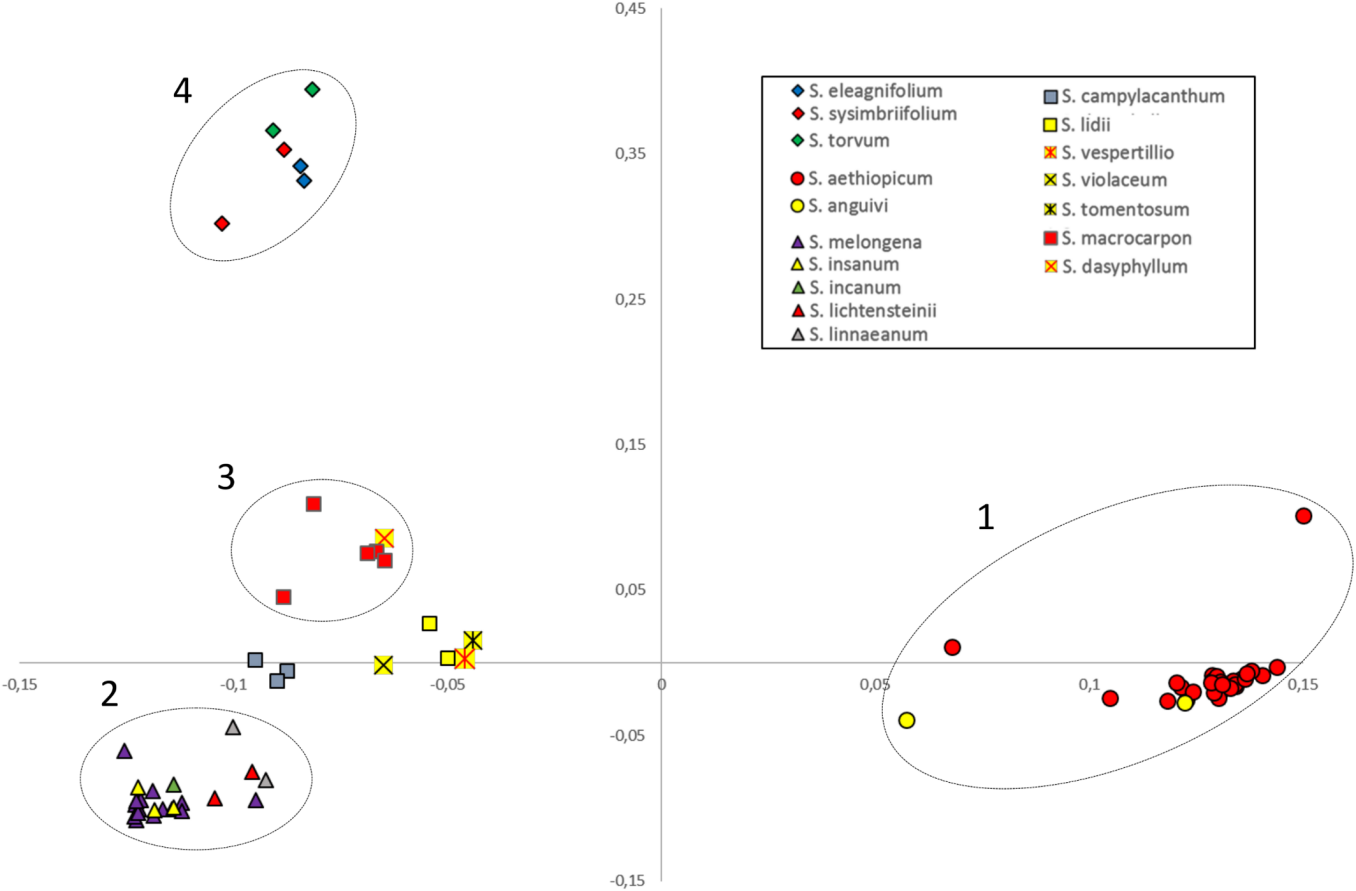
The genetic architecture of the full germplasm panel of 76 *Solanum* accessions. PCoA visualization of the genetic relationships within the full set of accessions.

The whole data set was also subjected to PCoA analysis (Fig 3) which, on the whole, confirmed the grouping of genotypes based on UPGMA and *FastSTRUCTURE* [48] clustering. Because a limited number of samples of each of the wild relatives was included in our study, the PCoA analysis did not allow to highlight the within-species diversity as it did in the cultivated species; however, it made possible some additional inferences. The first two axes explained 71.4% of the genetic variation. The first axis, explaining 57.6% of the genetic variation, clearly separated cultivated scarlet eggplant *S*. *aethiopicum* and its wild ancestor *S*. *anguivi* from all the other accessions, with no evident separate clustering of the two species. The latter are fully inter-fertile [34,51] and the identification of intermediate forms [27,29] suggests occurrence of genetic flow between them.

The second axis, explaining 13.8% of the genetic variation, clearly split the entries of *S*. *sisymbriifolium*, *S*. *torvum* and *S*. *elaeagnifolium*, which clustered in the previously described group 4, from the ones of sub-clusters 2 and 3 as well as the entries classified as admixed, i.e. brinjal and gboma eggplants, their respective progenitors *S*. *insanum* and *S*. *dasyphyllum* together with other Old World wild species, as well as the entries classified as admixed.

Both *S*. *sisymbriifolium* and *S*. *torvum* are native of South and Central America and, together with *S*. *viarum*, were classified in GP3 by Syfert et al. [30]. They have been also reported to be, within subgenus *Leptostemonum* (Dun.) Bitt., phylogenetically far away from the cultivated eggplants and the other Old World species [23,30,54,55]. *Solanum elaeagnifolium* is also a New World species [37] which was not included in the study of Syfert et al. [30], and whose origin is attributable to GP3 on the basis of the present results.

Both *S*. *sisymbriifolium* and *S*. *torvum* are of interest for eggplant breeding, as they are tolerant or resistant to many diseases [20]. Their high phylogenetic distance to cultivated eggplants is confirmed by the many ineffective attempts to hybridize them with *S*. *melongena* [29,56–58]. No sexual hybrids have ever been reported between *S*. *melongena* and *S*. *sisymbriifolium*, while interspecific hybrids obtained through embryo rescue of the cross *S*. *melongena* x *S*. *torvum* were highly sterile and no backcrosses have been reported to date [17]. Furthermore, although tetraploid somatic hybrids between either *S*. *sisymbriifolium* or *S*. *torvum* with *S*. *melongena* were obtained, they did not produce sexual offspring [59,60].

On the basis of PCoA analysis, the cultivated species which appears genetically closer to the cultivated eggplant is gboma eggplant (*S*. *macrocarpon*), clustering together with *S*. *dasyphyllum*, which has been reported by many authors to be its wild ancestor [23,35,52,54,61] (Fig 3). This seems to indicate that gboma eggplant, might be genetically closer to *S*. *melongena* than the cultivated scarlet eggplant (*S*. *aethiopicum*), which is included in section Oliganthes (Dunn.) Bit. [34,62]. However contrasting results have been reported in literature. Based on chloroplast DNA RFLPs [63], ISSRs [64], AFLPs and nrITS sequences [19] it was previously reported that *S*. *aethiopicum* is closer to *S*. *melongena* than *S*. *macrocarpon*; otherwise Sakata and Lester [65], in a study based on chloroplast DNA RFLPs, and Vorontosva et al. [23] using ITS, waxy and trnT-F regions sequences obtained opposite results. Interestingly, Furini and Wunder [66] using AFLPs as well as Levin et al. [54], Weese and Bohs [53] and Särkinen et al. [55] using several nuclear and plastid DNA sequences found that *S*. *aethiopicum* and *S*. *macrocarpon* were phylogenetically closer among them than to *S*. *melongena*. Studies based on the species inter-fertility highlighted that interspecific hybrids between *S*. *melongena* and *S*. *aethiopicum* as well as backcrosses could be easily obtained [17,67,68]; on the other hand, although hybrids between *S*. *melongena* and *S*. *macrocarpon* were obtained [56,67,69], in most cases they were high sterile and only the backcross of a tetraploid hybrid between the two species with *S*. *melongena* was successful [69]. The difficulty in obtaining the hybrids between these two species, despite being phylogenetically close [23,65], might be caused by some chromosomal rearrangement or other hybridization barriers. At last, Kouassi et al. [58] reported that the backcrosses towards *S*. *melongena* of the hybrid between *S*. *dasyphyllum* (wild ancestor of *S*. *macrocarpon*) and *S*. *melongena* was successful. A clarification is provided by our data obtained from *FastSTRUCTURE* analysis (Fig 2) which highlights that the three cultivated species belong to clearly separate groups, suggesting that *S*. *macrocarpon* should be excluded from section Melongena (Mill.) as proposed by Sakata et al. [63].

PCoA analysis also showed that *S*. *campylacanthum*, *S*. *incanum*, *S*. *insanum*, *S*. *lichtensteinii* and *S*. *linnaeanum*, which form part of the “brinjal eggplant” complex [52,53], cluster in proximity with eggplant (Fig 3). Among them, *S*. *campylacanthum* appears to be the most genetically differentiated from the others. This is in agreement with previous AFLP, nuclear and chloroplast DNA sequence results [23,53,61]. Indeed, interspecific hybrids were obtained between *S*. *campylacanthum* and *S*. *melongena*, but the number of seeded fruits and seeds per fruit was lower in respect to the ones obtained following crosses with other species within the “common eggplant” complex [52,58,70]. *Solanum linnaeanum* and the accession of *S*. *lichtensteinii* cluster together and close to *S*. *melongena*. This result confirms that the two species are genetically related [23,53] and supports the hypothesis that *S*. *linnaeanum* and *S*. *lichtensteinii* are of South African origin and share a common ancestor, although the former grows in several tropical and subtropical areas of the world [18,23].

*Solanum linnaeanum* and *S*. *lichtensteinii* produce hybrids with moderate or high fertility when crossed with eggplant [18], which can be also backcrossed with relative ease [17,19,23,58,28]. However our data show that they are genetically more distant from *S*. *melongena* than *S*. *incanum* or *S*. *insanum* [19,23,53,65,66]. *Solanum incanum* was suggested to be eggplant’s pre-domestication ancestor and is being used in eggplant breeding programs as a source of variation for phenolics content and resistance to drought [18]. Recent morphological and molecular work has shown that species-level differences exist between *S*. *incanum* and *S*. *melongena* and, on the basis of new evidence, *S*. *insanum* is considered the eggplant wild progenitor [36]. The two species are also fully inter-fertile and their hybrid produce many fruits and seeds [29]. It is also not surprising that, since frequent genetic flow occurs between both species in the indo-birmanian region [71,72], in our PCoA analysis the *S*. *insanum* accessions appear intermingled with the ones of *S*. *melongena*.

Our data show that the three species *S*. *lidii*, *S*. *tomentosum*, *S*. *vespertilio* cluster into proximity to each other and *S*. *violaceum* a little more apart (Fig 3). *Solanum lidii* and *S*. *vespertilio* are endemic to the Canary Islands (Spain) and are genetically similar sister species, which were found to cluster together in previous molecular studies [23,54,55,73,74]. In several molecular studies *S*. *tomentosum* was also found to cluster close to *S*. *lidii* and *S*. *vespertilio* [23,54,55,73], thus our results confirm that the three species are close relatives. *Solanum violaceum* clusters with these three taxa in both the *FastSTRUCTURE* and PCoA analyses in spite of having a native distribution in India and Southeast Asia [19].

### Within-groups PCoA analyses

**Fig 4:**
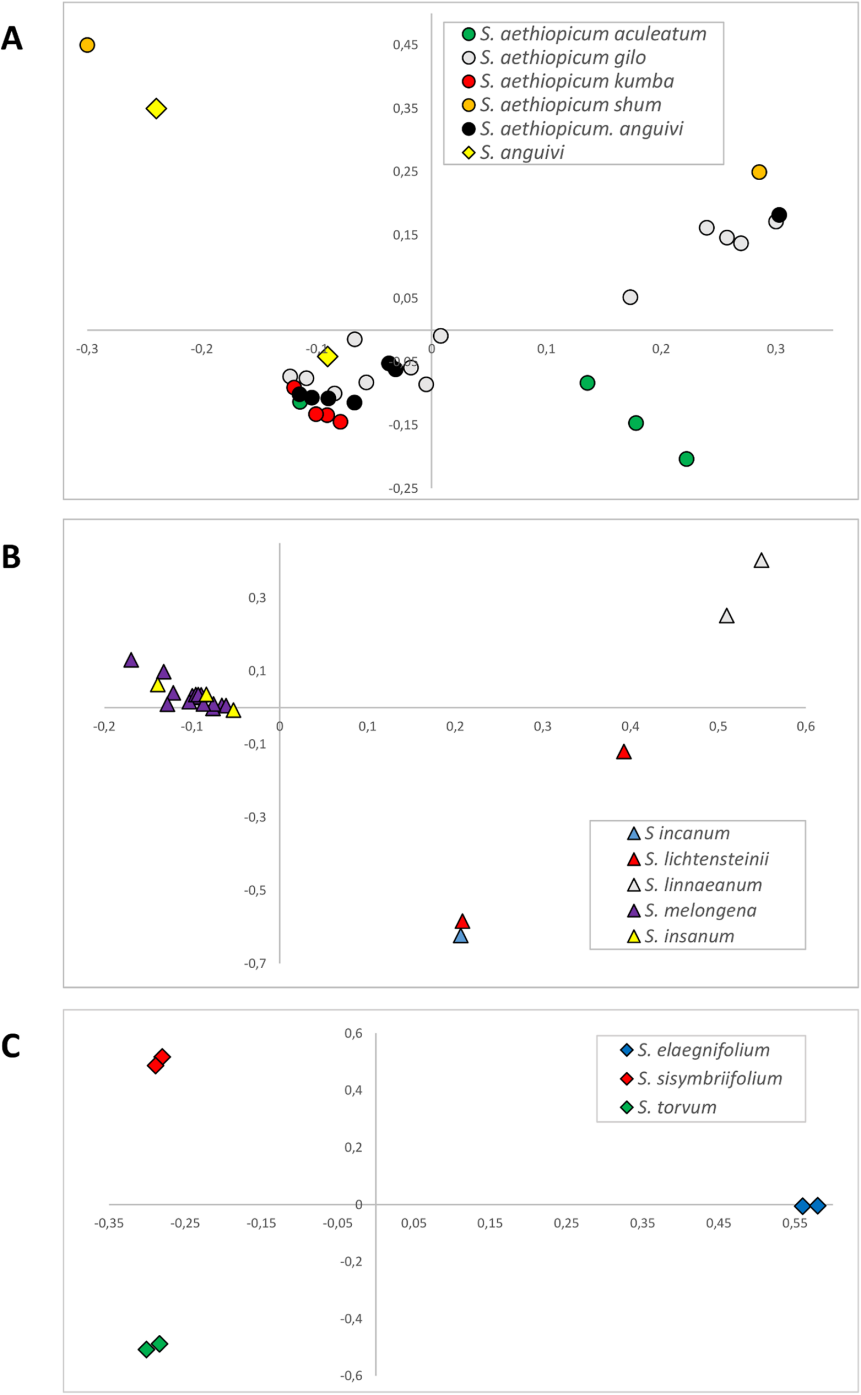
Within-groups PCoA analyses in subgroups of germplasm panel of *Solanum* accessions: visualization of the genetic relationships within sub-group 1 (A; scarlet eggplant complex), sub-group 2 (B; brinjal eggplant complex) and sub-group 4 (C; New World species).

In order to gain a better landscape of the genetic relationships among the species in study, PCoA analyses were also separately performed on entries clustering in the sub-groups 1, 2 and 4, following *FastSTRUCTURE* [48] analysis (Fig 4A, 4B and 4C). The separate PCoA of entries grouped in sub-group 1 (Fig 4A) confirmed that the different *S*. *aethiopicum* varietal types are partially intermingled and show a high within varietal type genetic diversity; furthermore, the absence of an evident genetic differentiation with their wild ancestor *S*. *anguivi* was confirmed. As observed by Sunseri et al. [24] in a molecular characterization based on AFLP and SSR markers, the different cultivar groups of *S*. *aethiopicum* were intermingled in the cluster analysis. The four cultivar groups (Aculeatum, Gilo, Kumba, and Shum) are distinguished by simple morphological traits, like fruit size and shape, fruit bitterness, and the presence or absence of prickles and star leaf hairs [26,34], which allow the differentiation among cultivars based on morphological characterizations [21]. However several of these traits, like prickliness and presence/absence of star leaf hair, seem to have a simple genetic basis in scarlet eggplant [51] while, as occurs in common eggplant [75,76], other traits (fruit size and shape) are under control of a few major genes. The genetic flow occurring between different groups, as a result of spontaneous or artificial hybridization, may thus result in a lack of (or reduced) genetic differentiation. Indeed, in a previous study [26], it was reported that the Aculeatum group seems to have been derived from hybridization between *S*. *aethiopicum* group Kumba and *S*. *anguivi*. On the whole, the varietal groups that showed the highest genetic differentiation were Aculeatum, (characterized by the highest anthocyanin content and prickliness in respect to all the others) and Shum, which differed for the mean average values of 8 of 18 morphological traits analysed in a previous study [21].

PCoA including accessions of the sub-group 2 (Fig 4B), as expected grouped separately the entries of eggplant and its wild relative *S*. *insanum* from the close relatives *S*. *incanum*, *S*. *lichtensteinii* and *S*. *linneanum*, the latter being the most genetically differentiated from all the others. The *S*. *melongena* accessions analysed included types, hailing from Sri Lanka, India and China as well as from Ivory Coast and Spain and producing fruits of different shape and colour. In a previous work [16] 191 eggplant accessions were scored for a set of 19 fruit and plant traits and the analysis of phenotypic data made it possible to classify the genotypes in three main fruit morphological groups producing: (i) elongated fruits, (mean ratio fs = fruit length/fruit maximum diameter around 5.05); (ii) semi-long fruits (fs from 1.2 to 2) and (iii) round fruits (fs around 1), which cut across the Oriental and Occidental divide. On the other hand STRUCTURE [77] analysis based on 24 microsatellite markers (22 genomic ones and two from EST), identified two major sub-groups, which to a large extent mirrored the provenance of the entries. In the present study, in spite of the wide set of polymorphisms detected, the accessions from different origin did not highlight a grouping together trend. This apparent discrepancy can be explained by either the difference in size of the two germplasm sets, but also by the number of markers applied, as the use of a limited number of selected markers might provide unrealistic estimates of genetic variability in the set of accessions in study.

PCoA including accessions of the sub-group 4 (Fig 4C) highlights that *S*. *sisymbriifolium*, *S*. *torvum* and *S*. *elaeagnifolium* are genetically far away from each other and that their grouping in the sub-group 4 is due to their common high genetic divergence from all the other entries. This is confirmed by previous molecular results that includes *S*. *torvum* and *S*. *sisymbriifolium* in different clades within subgenus Leptostemonum from the cultivated eggplants [23,30,54,55]. Furthermore, on the basis of PCoA analysis, the two accessions of *S*. *torvum* form a group 'per se' in respect to all the others.

Previous phylogenetic studies placed *S*. *elaeagnifolium* and the rest of species of the Elaeagnifolium clade closer to Old World species than either *S*. *sisymbriifolium* or *S*. *torvum* [19,23,54,55,73]. Recently, crossing data confirm that *S*. *elaeagnifolium* is closer to eggplant than either *S*. *sisymbriifolium* and *S*. *torvum*, as interspecific hybrids have been obtained which present intermediate fertility [58], and with which it is possible to obtain backcrosses with *S*. *melongena* (unpublished results).

## Conclusions

One of the most exciting developments in the past decade has been the application of powerful and ultra-rapid nucleic acid sequencing techniques to the study of genetic relationships and phylogeny of crop species [78]. As previously reported by Bajaj et al. [79] in chickpea, our results demonstrate that the high-throughput genotyping of numerous genome-wide SNP markers represents a highly and more effective approach, in respect to the ones based on limited sets of genome-wide markers or a small set of gene sequences, for understanding the extent of natural allelic diversity and genetic relationships among and within wild and cultivated species belonging to eggplant complexes. The high number of detected polymorphisms were analysed by *FastSTRUCTURE* [48, 49], UPGMA and PCoA analyses and the three approaches showed to be complementary in the interpretation of data. On the whole, we confirm a wide genetic base and broad molecular diversity among wild and cultivated species within and among the three cultivated eggplant complexes and the New World eggplant CWRs. Thanks to a reduced complexity genome sequencing approach, we were able to fingerprint all accessions in the study and gathered information which may efficiently guide further exploration of the diversity and relationships in the large *Solanum* subgenus *Leptostemonum* group. The approach used and data obtained lay the foundation also to address the evaluation of gene flow among inter-fertile sympatric taxa [71], recent speciation and domestication processes of cultivated eggplants. In addition, the large number of markers distributed across the genome may also contribute to facilitate the transfer of target genomic regions controlling useful agronomic traits, such as biotic and abiotic stress tolerance or fruit quality traits, from related species into the genetic background of cultivated eggplants.

## Supporting information

S1 Table SNPs detected in the genome and in CDSs. In both cases, number and percentage of: (i) missing sites; (ii) detected SNPs, the percentage is evaluated as the ratio between detected SNPs/Genomic or CDS total SNPs-missing sites; (iii) heterozygous SNPs, the percentage is evaluated as the ratio between heterozygous SNPs/ Genomic or CDS total SNPs-missing sites; (iv) homozygous SNPs, the percentage is evaluated as the ratio between homoyigous SNPs/ Genomic or CDS total SNPs-missing sites.
(XLSX)

S1 Fig Distribution of sequenced reads, after quality cleaning and trimming procedures, across a germplasm panel of 76 *Solanum* accessions (in million reads). (TIFF)

S2 Fig *FastSTRUCTURE* output at K = 4 from full germplasm panel together with outputs of separate analyses performed with subsets of taxa. Asterisks indicate the best K choice based on the ΔK method.
(TIF)
