# Supplementary figures and images for: Coding SNPs analysis highlights genetic relationships and evolution pattern in eggplant complexes
Source: PLoS One. 2017 Jul 7;12(7):e0180774. doi: 10.1371/journal.pone.0180774 (PMC5501601; doi:10.1371/journal.pone.0180774)

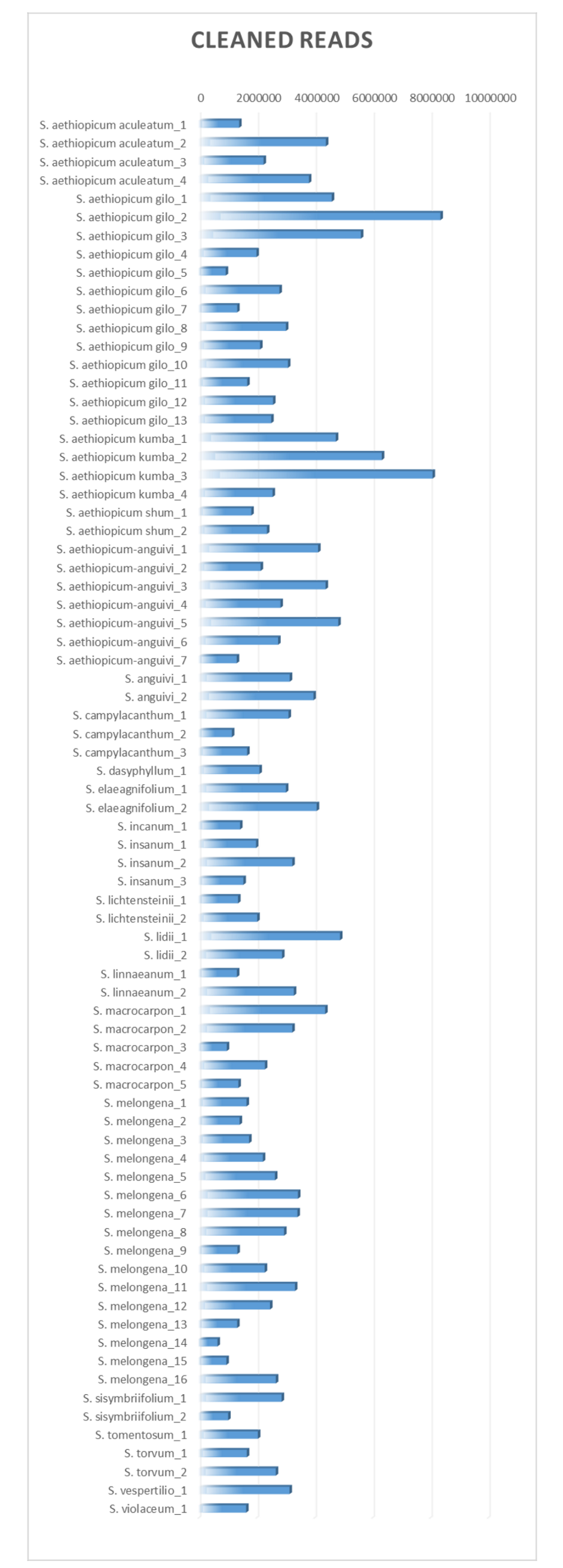

Supplement: S1 Fig — (TIFF) [file pone.0180774.s002.tiff]

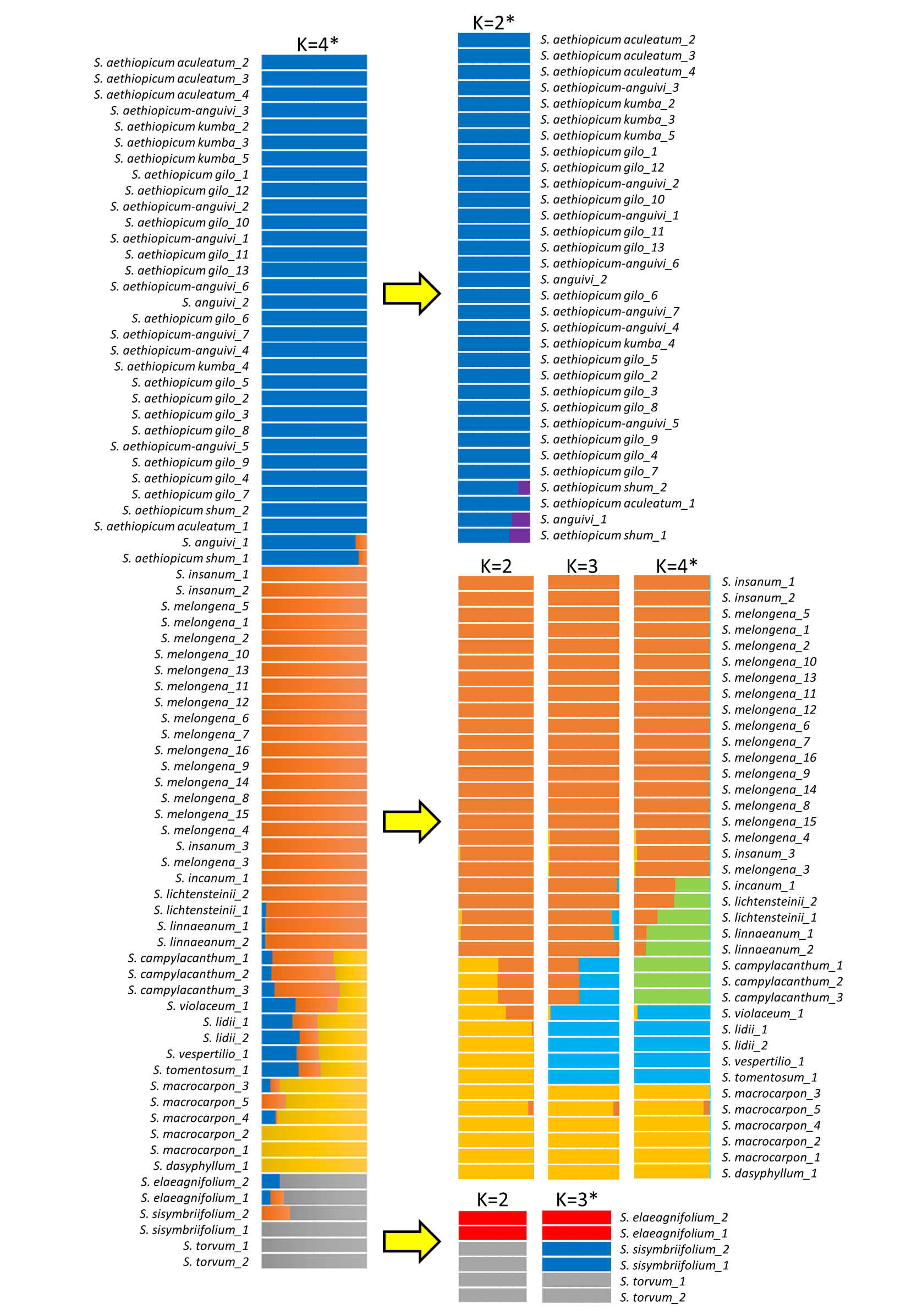

Supplement: S2 Fig — Asterisks indicate the best K choice based on the ΔK method. (TIF) [file pone.0180774.s003.tif]
